# Web-Based Return of Individual Patient-Reported Outcome Results Among Patients With Lymphoma: Randomized Controlled Trial

**DOI:** 10.2196/27886

**Published:** 2021-12-14

**Authors:** Simone Oerlemans, Lindy Paulina Johanna Arts, Jacobien M Kieffer, Judith Prins, Mels Hoogendoorn, Marjolein van der Poel, Ad Koster, Chantal Lensen, Wendy Bernadina Catharina Stevens, Djamila Issa, Johannes F M Pruijt, Margriet Oosterveld, René van der Griend, Marten Nijziel, Lidwine Tick, Eduardus F M Posthuma, Lonneke V van de Poll-Franse

**Affiliations:** 1 Department of Research and Development Netherlands Comprehensive Cancer Organisation Utrecht Netherlands; 2 Division of Psychosocial Research and Epidemiology Netherlands Cancer Institute Amsterdam Netherlands; 3 Department of Medical Psychology Radboud University Medical Center Nijmegen Netherlands; 4 Department of Hematology Medical Center Leeuwarden Leeuwarden Netherlands; 5 Department of Internal Medicine, Division of Hematology GROW School for Oncology and Developmental Biology Maastricht University Medical Centre Maastricht Netherlands; 6 Department of Internal Medicine VieCuri Medical Centre Venlo/Venray Netherlands; 7 Department of Internal Medicine Bernhoven Hospital Uden Netherlands; 8 Department of Hematology Radboud University Medical Center Nijmegen Netherlands; 9 Department of Internal Medicine Jeroen Bosch Hospital 's-Hertogenbosch Netherlands; 10 Department of Internal Medicine Canisius-Wilhelmina Hospital Nijmegen Netherlands; 11 Department of Internal Medicine Diakonessenhuis Utrecht/Zeist Netherlands; 12 Department of Internal Medicine Catharina Hospital Eindhoven Netherlands; 13 Department of Internal Medicine Máxima Medical Centre Veldhoven Netherlands; 14 Department of Internal Medicine Reinier de Graaf Group Delft Netherlands; 15 Department of Internal Medicine Leiden University Medical Center Leiden Netherlands; 16 Center of Research on Psychological and Somatic disorders Department of Medical and Clinical Psychology Tilburg University Tilburg Netherlands

**Keywords:** lymphoma, patient-reported outcomes, return of individual results, randomized controlled trial, self-management

## Abstract

**Background:**

There has been a cultural shift toward patient engagement in health, with a growing demand from patients to access their results.

**Objective:**

The Lymphoma Intervention (LIVE) trial is conducted to examine the impact of return of individual patient-reported outcome (PRO) results and a web-based self-management intervention on psychological distress, self-management, satisfaction with information, and health care use in a population-based setting.

**Methods:**

Return of PRO results included comparison with age- and sex-matched peers and was built into the Patient-Reported Outcomes Following Initial Treatment and Long-Term Evaluation of Survivorship registry. The self-management intervention is an adaptation of a fully automated evidence-based intervention for breast cancer survivors. Patients with lymphoma who completed the web-based questionnaire were equally randomized to care as usual, return of PRO results, and return of PRO results plus self-management intervention. Patients completed questionnaires 9 to 18 months after diagnosis (T0; n=227), 4 months (T1; n=190), 12 months (T2; n=170), and 24 months (T3; n=98).

**Results:**

Of all invited patients, 51.1% (456/892) responded and web-based participants (n=227) were randomly assigned to care as usual (n=76), return of PRO results (n=74), or return of PRO results and access to Living with lymphoma (n=77). Return of PRO results was viewed by 76.7% (115/150) of those with access. No statistically significant differences were observed for psychological distress, self-management, satisfaction with information provision, and health care use between patients who received PRO results and those who did not (*P*>.05). Use of the self-management intervention was low (2/76, 3%), and an effect could therefore not be determined.

**Conclusions:**

Return of individual PRO results seems to meet patients’ wishes but had no beneficial effects on patient outcome. No negative effects were found when individual PRO results were disclosed, and the return of individual PRO results can therefore be safely implemented in daily clinical practice.

**Trial Registration:**

Netherlands Trial Register NTR5953; https://www.trialregister.nl/trial/5790

**International Registered Report Identifier (IRRID):**

RR2-10.1186/s13063-017-1943-2

## Introduction

### Background

Patients with lymphoma are at risk of experiencing adverse effects of cancer and its treatment, such as fatigue, cognitive problems, and neuropathy [1-4]. In addition, up to a quarter experience persistent levels of anxiety, depressive symptoms, and fear [5], also known as psychological distress. Both symptoms and psychological distress may be exacerbated when information and supportive care are unavailable [6,7]. This may, in turn, lead to increased health care use [8]. Regular screening of physical and psychosocial symptoms using patient-reported outcomes (PROs) could increase awareness and recognition of symptoms and can contribute to their management [9-13].

Since the past decade, there has been a cultural shift toward patient engagement in health, with a growing demand from patients to access their individual results [14-17]. Returning individual PRO results enables patients to monitor their functioning and create awareness of symptoms. Furthermore, it offers patients the opportunity to compare their scores with peers [15] to evaluate if their scores are *normal* and to incorporate this information into personal decision-making [16]. However, some clinicians have expressed reservations about disclosing PRO research results to patients, as it may cause patients to become more concerned and lead to increased health care use by patients and higher workload for clinicians. Therefore, it is important to investigate whether patients will be in a better or worse condition with their PRO results being disclosed and which patients wish to receive their PRO results.

To improve health outcomes, subsequent steps such as self-management interventions are expected to be necessary in addition to monitoring and returning PRO results [18]. Self-management interventions intend to enhance patients’ knowledge and skills and empower them to play an active role in the management of their disease and its consequences [19,20]. Studies on the outcomes of self-management are mainly based on patients with solid cancers and are not consistent, with some randomized controlled trials (RCTs) showing no effects [21-23]. In addition, most self-management interventions are not specifically aimed at patients with lymphoma and are found to be effective among selected groups of patients. Little is known about the effects of such interventions in a population-based setting within an unselected group of patients.

### Objectives

To investigate the effect of (1) return of individual PRO research results, including comparison with peers [24], and (2) a web-based self-management intervention Living with lymphoma, the Lymphoma Intervention (LIVE) trial was performed. The primary objective of the LIVE RCT was to examine the effects of return of PRO results to patients with or without access to Living with lymphoma on self-management, satisfaction with information, and psychological distress in a population-based setting of patients with lymphoma [24]. We hypothesized that those with access to the return of PRO results or access to LIVE would have higher levels of self-management and satisfaction with information and lower levels of psychological distress. On the basis of new insights that psychologically distressed patients reported increased health care use [8], we also investigated the effects of return of PRO results to patients with or without access to Living with lymphoma on health care use (secondary objective). Furthermore, we explored sociodemographic, clinical, and psychological differences between patients who viewed their individual PRO results and those who did not.

## Methods

### Design and Participants

This RCT was embedded in the population-based Patient-Reported Outcomes Following Initial Treatment and Long-Term Evaluation of Survivorship (PROFILES) registry [25]. PROFILES enables PRO data collection management and links PRO data to clinical data from the Netherlands Cancer Registry (NCR).

Between October 2015 and February 2019, patients diagnosed with lymphoma (Hodgkin lymphoma, non-Hodgkin lymphoma, or chronic lymphocytic leukemia), as defined by the International Classification of Diseases for Oncology-3 codes [26], from 13 hospitals in the Netherlands were selected for participation 9 to 18 months after diagnosis. The NCR registers all newly diagnosed patients with cancer in the Netherlands within the first year after diagnosis and routinely collects detailed data on sociodemographic and clinical characteristics (eg, age and sex, date of diagnosis, cancer type, and primary treatment). Treating hemato-oncologists were asked to verify the eligibility of the patients. As we aimed to keep a population-based approach, we only defined a few exclusion criteria, such as presence of severe psychopathology or dementia, being in transition to terminal care, and not being able to complete a Dutch questionnaire. Patients were informed that completion of the web-based questionnaire resulted in RCT enrollment with automatic randomization to 1 of the 3 study arms. Paper respondents were not eligible for the RCT (as the return of PRO results and *Living with lymphoma* were web-based) and were observationally followed within the PROFILES lymphoma registry. A reminder mail was sent after 3 weeks. Respondents received follow-up questionnaires at 4 months (T1), 12 months (T2), and 24 months (T3) after the baseline questionnaire. More details about the study design, enrollment, and sample size calculation have previously been published in the research protocol [24]. The RCT was centrally and locally approved by a medical research ethics committee [24].

### Randomization

Randomization was performed using block randomization to ensure a balance in sample size across arms over time [27]. Participants were equally randomized to (1) care as usual (CAU), (2) CAU plus return of PRO results, or (3) CAU plus return of PRO results and Living with lymphoma.

### Interventions Versus CAU

#### Arm 1: CAU

In arm 1, patients received CAU from their hemato-oncologists and oncology nurses. In general, they provided verbal information to their patients and provided leaflets regarding the diagnosis and treatment they received.

#### Arm 2: Return of PRO Results

In arms 2 and 3, in addition to CAU, individual PRO research results were disclosed to patients. This feedback was automatically generated after the completion of the questionnaire. On the basis of respect for autonomy, patients had the choice as to whether they wanted to receive the results [14] and could click on the *feedback* button for return of results.

Detailed information about the return of PRO results (also known as PRO feedback) has been described elsewhere [15,24]. In short, individual research results on general health-related quality of life (HRQoL), physical, emotional, cognitive, and social functioning, fatigue, neuropathy, anxiety, and depressive symptoms were returned to patients [24]. Individual scores were integrated into graphical displays with colored bar charts [28,29]. Patients had the opportunity to compare their scores to mean scores of other patients with lymphoma and an age- and sex-matched normative population without cancer [30] to determine whether their scores were average or not. The colors of the bar charts were related to clinically relevantly mean differences of the evidence-based guidelines of the European Organisation for Research and Treatment of Cancer Quality of Life Questionnaire Organization [31] and considered *average* (amber); *above average* (green); or *below average* (red). Patients with above-average symptom scores were advised to contact their general practitioner.

#### Arm 3: Return of PRO Results + Living With Lymphoma

In addition to the return of PRO results, patients in arm 3 had access to a web-based self-management intervention—Living with lymphoma. A detailed description of Living with lymphoma has been described elsewhere [24]. Living with lymphoma, an adaptation of the evidence-based BREATH (Breast Cancer eHealth) intervention for breast cancer [32,33], was based on psychoeducation and cognitive behavioral therapy techniques to enhance patients’ knowledge and skills. The intervention also included a library with background and additional information on various subjects (eg, work, sexuality, lifestyle) and reference to additional health care services (eg, psychologists, physiotherapists). It was left to the discretion of the patients how and to what extent they used the intervention. The intervention was fully automated, nonguided, and delivered without professional therapist support.

### Measures

#### Sociodemographic and Clinical Measures

Sociodemographic characteristics (age and sex) and detailed clinical information (date of diagnosis, cancer type, and primary treatment) were obtained from the NCR. NCR data were available for both RCT participants and nonparticipants. Information on educational level and marital status was assessed in the questionnaire (data only available for RCT participants).

Comorbidities at the time of questionnaire completion were assessed using an adapted version of the Self-Administered Comorbidity Questionnaire [34]. Patients were asked to identify comorbidities present within the past 12 months—heart disease, hypertension, arthritis, stroke, lung disease, diabetes, stomach disease, kidney disease, liver disease, anemia, thyroid disease, and rheumatoid arthritis. Positive responses were summed to a total score ranging from 0 to 12 (data only available for RCT participants).

#### Psychological Measures

Personality traits were assessed using the Big Five Inventory [35], a 44-item inventory for measuring the Big Five personality traits—neuroticism, extraversion, openness to experience, agreeableness, and conscientiousness. Items are scored on a 5-point scale. Scale scores were obtained by averaging all items for each trait and range from 0 to 5 [36].

The 40-item Mental Adjustment to Cancer scale was used to assess adjustment to cancer in terms of coping strategies [37,38]. Items were grouped on five categories: *Helplessness/Hopelessness*, *Anxious Preoccupation*, *Fighting Spirit*, *Fatalism*, and *Avoidance*. Each item is rated on a 4-point scale. Scale scores were obtained by averaging all items of each strategy and range from 1 to 4. Higher scores represent higher endorsement of the coping strategy.

Health-related quality of life was assessed using the 30-item Quality of Life Questionnaire from the European Organisation for Research and Treatment of Cancer [39]. This questionnaire includes five functional scales, three symptom scales, a global health and quality of life scale, and several single-item symptom measures. Items are scored on a 4-point Likert scale, except for the global health and quality of life scale that is scored on a 7-point linear analog scale. After linear transformation, all scales and single item measures range in score from 0 to 100. Higher scores on functional and health and quality of life scales indicate better functioning or HRQoL, whereas higher scores on symptom scales indicate more complaints.

#### Self-management Skills

Self-management skills were assessed using the Health Education Impact Questionnaire, which contains 40 items across 8 scales—positive and active engagement in life, health-directed activities, skill and technique acquisition, constructive attitudes and approaches, self-monitoring and insight, health service navigation, social integration and support, and emotional distress [40]. Each item was scored on a 4-point scale. Scale scores were obtained by averaging all items of each domain and ranged from 1 to 4. Higher scores indicate better status or self-management, except for emotional distress, in which higher scores indicate higher distress [40].

#### Satisfaction With Information Provision

Satisfaction with overall information provision was assessed using an adapted version of the 9-item Information Satisfaction Questionnaire [41]. Patients were also asked to rate their level of satisfaction with overall information on a scale that ranged from 1 (“very unsatisfied”) to 5 (“very satisfied”). In addition, information on patient information preferences was available. Patients were asked to categorize themselves into those who would like (1) all available information, (2) only positive information, and (3) only limited information about their disease. Finally, patients had to categorize themselves into those who would like (1) to be involved in the decision-making process about their disease, or (2) the physician to make the decisions.

#### Psychological Distress

Psychological distress was assessed using the 14-item Hospital Anxiety and Depression Scale [42]. Each item was rated on a 4-point scale ranging from 0 to 3. The sum score was obtained by adding all item scores and ranged from 0 to 42. Higher scores indicated higher levels of psychological distress [43]. Patients with a Hospital Anxiety and Depression Scale sum score ≥13 were categorized as *psychologically distressed* [44].

#### Health Care Use

Two open questions were asked to assess health care use: (1) “How often did you contact a general practitioner in the past 12 months?” and (2) “How often did you visit a medical specialist in the past 12 months?”

### Statistical Analyses

#### Overview

Sociodemographic and clinical characteristics between the three arms were compared using univariable analyses of variance and chi-square tests. If at least half of the items from a subscale were completed, the missing items were replaced by the average of those that were present for the participant.

To model between-group differences in change from baseline (T0) to follow-up (T1-T3), mixed-effects models were used with an unstructured covariance structure and a restricted maximum likelihood solution [45]. A random intercept at the patient level was included to adjust for interdependency between repeated measures. The CAU-arm (arm 1) was assigned as the reference group. The *P* value for overall model effects was set at .05, and for specific contrasts at .01, lowering the risk of type I errors as a result of multiple testing. In the iterative process of variable selection, a priori selected covariates (age, sex, cancer type, and treatment) were removed from the model as they were nonsignificant and had no confounders. However, as those in the CAU arm seemed to be somewhat more often psychologically distressed (17/77, 22%) than patients in the return of results arm (9/74, 12%) and the arm with return of results and access to Living with lymphoma (8/76, 11%; *P*=.10; Table 1), we considered psychological distress as a confounding factor and adjusted for baseline psychological distress in analyses (when psychological distress was not the outcome variable). Group differences in mean change scores from baseline to follow-up were accompanied by Cohen effect size (ES). Cohen ES was calculated by dividing the difference in mean change scores between the control and intervention groups by the pooled baseline SD. An ES of 0.20 was considered small, 0.50 moderate, and 0.80 large [44,46]. All analyses were conducted on an intention-to-treat basis. All statistical analyses were performed using SAS version 9.4.

**Table 1:** Baseline characteristics of participants according to randomized controlled trial (RCT) arm and of nonparticipants.

				CAU^a^ (n=77)		Return of PRO^b^ results (n=74)		Return of PRO results + living with lymphoma (n=76)		*P* value^c^			Total RCT participants (n=227)			Nonparticipants (n=666)			*P* value^d^	
**Sociodemographic** **characteristics**																				
	Age at time of questionnaire (years), mean (SD)			61.3 (12.9)		60.0 (13.4)		60.8 (14.0)		.83			60.7 (13.4)			65.3 (15.7)			<.001	
	**Sex, n (%)**										.73									<.001
		Male	53 (68.8)		55 (74.3)		53 (69.7)					161 (70.9)			377 (56.6)					
		Female	24 (31.2)		19 (25.7)		23 (30.3)					66 (29.1)			289 (43.4)					
	**Educational level^e^, n (%)**										.71									—^g^
		Low	1 (1.3)		2 (2.7)		3 (3.9)					6 (2.6)			N/A^f^					
		Medium	33 (42.9)		38 (51.4)		35 (46.1)					106 (46.7)			N/A					
		High	42 (54.5)		34 (45.9)		38 (50)					114 (50.2)			N/A					
	Partner (yes), n (%)			62 (80.5)		65 (87.8)		63 (82.9)		.46			190 (83.7)			N/A			—	
**Clinical characteristics**																				
	Months since diagnosis: mean (SD)			14.0 (3.1)		14.0 (3.6)		13.9 (3.0)		.94			14.0 (3.2)			14.0 (3.5)			.80	
	**Cancer type, n (%)**																			
		Hodgkin lymphoma	10 (12.9)		8 (10.8)		9 (11.8)		.99			27 (11.9)			75 (11.3)			.95		
		NHL-HG^h^	41 (53.2)		41 (55.4)		42 (55.3)					125 (55.1)			359 (53.9)					
		NHL-LG^i^	18 (23.4)		19 (25.7)		19 (25)					56 (24.7)			169 (25.4)					
		CLL^j^	8 (10.4)		6 (8.1)		6 (7.9)					19 (8.4)			63 (9.5)					
	**Primary treatment, n (%)**										.10									.13
		Active surveillance	23 (29.9)		18 (24.3)		12 (15.8)					53 (23.3)			199 (28.5)					
		Received active treatment	52 (67.5)		56 (75.7)		64 (84.2)					172 (75.8)			458 (68.8)					
		Chemotherapy	37 (48.1)		46 (62.2)		54 (71.1)					137 (60.4)			357 (54)					
		Radiotherapy	5 (6.5)		4 (5.4)		3 (3.9)					12 (5.3)			60 (8.1)					
		Stem cell transplantation	8 (10.4)		3 (4.1)		3 (3.9)					14 (6.2)			4 (0.6)					
		Other	2 (2.6)		3 (4.1)		4 (5.3)					9 (3.9)			37 (5.6)					
	**Ann Arbor stage**										.81									.30
		Stage I, n (%)	6 (7.8)		8 (10.8)		15 (19.7)					29 (12.8)			112 (16.8)					
		Stage II, n (%)	8 (10.4)		13 (17.6)		15 (19.7)					36 (15.8)			95 (14.3)					
		Stage III, n (%)	11 (14.3)		9 (12.2)		10 (13.2)					30 (13.2)			97 (14.6)					
		Stage IV, n (%)	30 (38.9)		30 (40.5)		26 (34.2)					86 (37.9)			194 (29.1)					
		Not determined (CLL) or missing, n (%)	22 (28.6)		14 (18.9)		10 (13.2)					46 (20.3)			168 (25.2)					
		Number of self-reported comorbidities: mean (SD)	1.3 (1.2)		1.2 (1.1)		1.0 (1.0)		.22			1.1 (1.1)			N/A					
**Psychological characteristics, n (%)**																				
	Psychological distress			17 (22.1)		9 (12.2)		8 (10.5)		.09			34 (14.9)			N/A			—	

^a^CAU: care as usual.

^b^PRO: patient-reported outcome.

^c^Reports comparisons between the intervention arms and the care as usual arm.

^d^Reports comparisons between randomized controlled trial participants and nonparticipants.

^e^Education levels were low=none or primary school; medium=lower general secondary education or vocational training; or high=preuniversity education or high-level vocational training or university.

^f^N/A: not applicable.

^g^Not available.

^h^NHL-HG: high-grade non-Hodgkin lymphoma.

^i^NHL-LG: low-grade non-Hodgkin lymphoma.

^j^CLL: chronic lymphocytic leukemia.

#### Power Calculation

With more than 74 participants per study arm, the study had 90% power to detect an ES of 0.50 with a two-tailed *P* value set at .05 [47].

## Results

### Baseline Characteristics of RCT Participants

**Figure 1:**
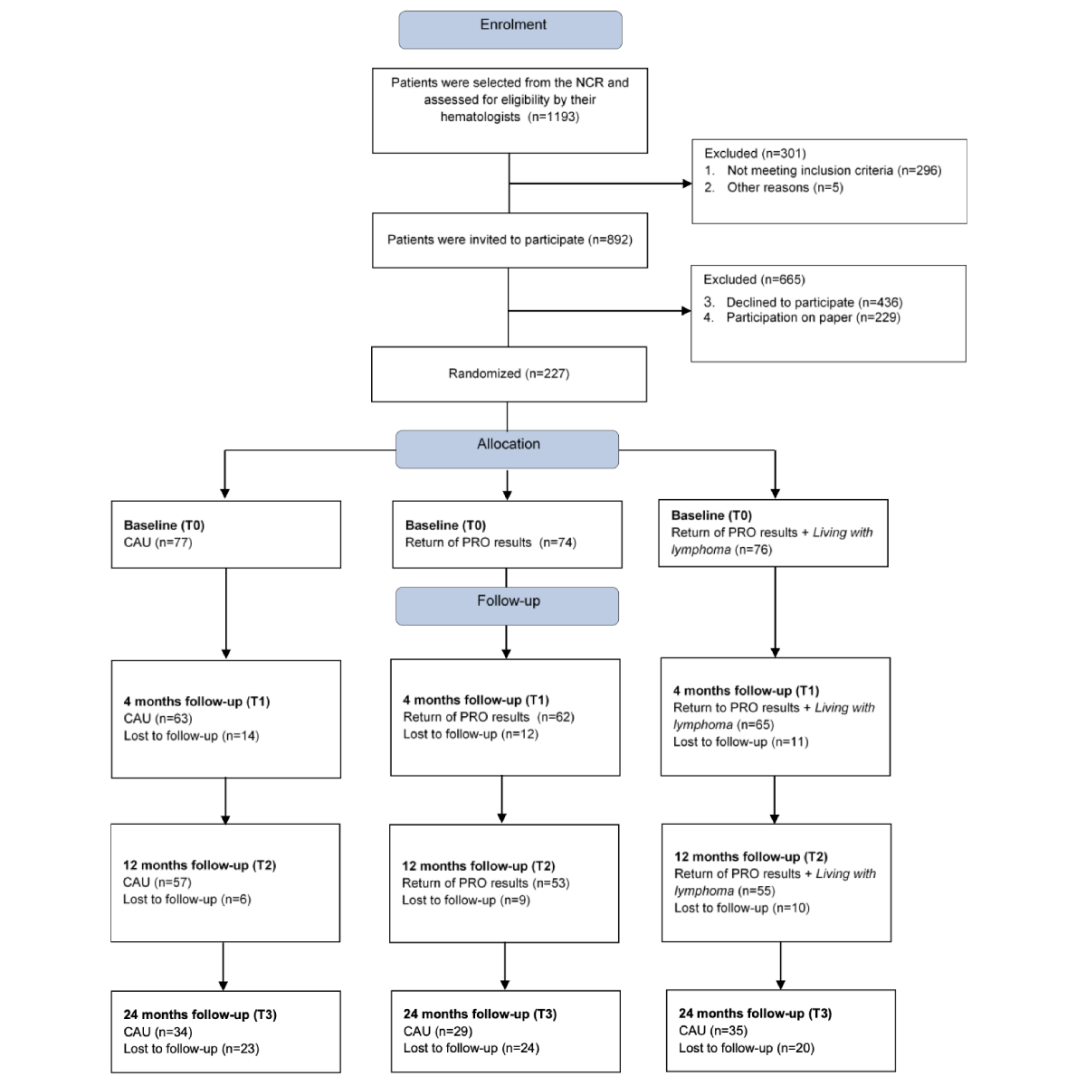
CONSORT (Consolidated Standards of Reporting Trials) flow diagram of the progress of the patients with lymphoma through the phases of the Lymphoma Intervention (LIVE) trial. CAU: care as usual (control group); NCR: Netherlands Cancer Registry; PRO: patient-reported outcome.

In total, 1193 patients were selected from the NCR and 892 patients were invited to participate. Of these, 51.1% (456/892) participated, of which 25.7% (229/892) were excluded for the RCT as they completed the questionnaire on paper (CONSORT [Consolidated Standards of Reporting Trials] diagram; Figure 1).

Overall, 25.4% (227/892) completed the web-based questionnaire and were included in the RCT. They were randomly assigned to CAU (control group; n=76), return of PRO results (n=74), or return of PRO results and access to Living with lymphoma (n=77). Completion rates of follow-up questionnaires were 84.1% (191/227) on T1, 74.9% (143/191) on T2, and 68.5% (98/143) on T3, and did not differ significantly among groups.

Those who declined participation or completed the questionnaire on paper were analyzed as nonparticipants in this study. All participants provided written informed consent.

RCT participants were younger than nonparticipants (60.7 vs 65.3 years; *P*<.001), and more often men (161/227, 70.9% vs 377/666, 56.6%; *P*<.001). RCT participants were on average 14.0 months after diagnosis (SD 3.2 months). The majority of RCT participants had a partner (190/227, 83.7%). Of the RCT participants, 75.8% (172/227) received active treatment, mostly chemotherapy (137/227, 60.4%), whereas 23.3% (53/227) were on active surveillance. The majority of patients had Ann Arbor stage IV disease at the time of diagnosis. Other baseline sociodemographic and clinical characteristics did not differ across groups (Table 1).

### The Living With Lymphoma Self-management Intervention

The use of self-management intervention was very low (3/76, 4%). Therefore, the effects of *Living with lymphoma* could not be determined within this RCT and were not included in the results. The analyses were performed with the three original RCT arms to maintain a power of 90%.

As we observed that adherence to Living with lymphoma intervention was very low one year after starting patient inclusion, research assistants sent an email for inquiry. A random sample of 5 patients who had access to the intervention and signed up were asked to respond without any obligation and were asked for their reasons for nonadherence. Two patients responded that they felt well and still had regular appointments with their hematologist, and therefore were not in need of an intervention:

In the first place, I feel very well, both physically and mentally. Secondly, I have regular appointments with my treating haematologist. Furthermore, I do not always want to be confronted with my disease.

Male, 79 years

I am not really concerned about the fact that I have had cancer. I am in remission for one year now, and that is how I feel. Every three months, I still have checkup appointments, but other than that I live my life the way I did before I had cancer. I feel fine, I have no limitations, and therefore I do not need information about a disease from the past.

Male, 54 years

### Return of Individual PRO Results

No statistically significant differences were observed for psychological distress, self-management subscales, and satisfaction with information provision between patients who received their individual PRO results and those who did not (Table 2). In addition, the return of PRO results did not have a significant effect on health care use. As no significant overall group-by-time interaction was found for the outcome variables, we were not allowed to explore specific contrasts.

**Table 2:** Between-group differences in mean change from baseline to follow-up.

			T0				T1					T2					T3					Between-group difference, T0-T1					Between-group difference, T0-T2					Between-group difference, T0-T3		
			n		Value, mean (SD)		n		Value, mean (SD)		n			Value, mean (SD)		n			Value, mean (SD)		Value, mean change (SE)			*P* value		Value, mean change (SE)			*P* value		Value, mean change (SE)			*P* value
**Psychological distress (HADS^a^ total; *P*=.94^b^)**																																		
	CAU^c^	77		7.03 (7.10)		62		6.98 (7.48)		54			6.06 (6.09)		34			6.94 (6.70)		N/A^d^			N/A		N/A			N/A		N/A			N/A	
	Return of PRO^e^ results	74		6.59 (5.26)		60		6.43 (6.21)		53			6.18 (5.61)		28			7.11 (8.26)		0.04 (0.68)			.96		0.20 (0.71)			.77		–0.71 (0.88)			.42	
	Return of PRO results + *Living with lymphoma*	76		5.75 (5.04)		64		5.78 (4.99)		59			5.68 (5.62)		35			5.34 (5.78)		0.02 (0.67)			.98		0.12 (0.69)			.86		–0.87 (0.83)			.30	
**Self-management skills (HeiQ positive and active engagement in life^f^; *P*=.52^b^)**																																		
	CAU	76		3.16 (0.52)		62		3.15 (0.52)		53			3.25 (0.49)		34			3.16 (0.48)		N/A			N/A		N/A			N/A		N/A			N/A	
	Return of PRO results	74		3.22 (0.46)		62		2.23 (0.50)		53			3.23 (0.51)		29			3.17 (0.65)		0.02 (0.07)			.81		–0.11 (0.07)			.12		–0.04 (0.09)			.66	
	Return of PRO results + *Living with lymphoma*	76		3.22 (0.44)		64		3.26 (0.52)		59			3.22 (0.48)		35			3.24 (0.41)		0.07 (0.07)			.34		–0.07 (0.07)			.33		0.02 (0.09)			.82	
**HeiQ health-directed behavior (*P*=.80^b^)**																																		
	CAU	77		3.34 (0.62)		62		3.28 (0.56)		55			3.35 (0.64)		34			3.16 (0.57)		N/A			N/A		N/A			N/A		N/A			N/A	
	Return of PRO results	74		3.27 (0.53)		62		3.29 (0.59)		53			3.28 (0.49)		29			3.19 (0.47)		0.08 (0.09)			.33		0.00 (0.09)			.69		0.15 (0.11)			.19	
	Return of PRO results + *Living with lymphoma*	76		3.22 (0.58)		64		3.23 (0.65)		59			3.26 (0.63)		35			3.21 (0.66)		0.09 (0.09)			.27		0.04 (0.09)			.97		0.12 (0.11)			.28	
**HeiQ skill and technique acquisition (*P*=.90^b^)**																																		
	CAU	76		2.94 (0.53)		62		2.97 (0.46)		53			3.02 (0.49)		34			3.05 (0.45)		N/A			N/A		N/A			N/A		N/A			N/A	
	Return of PRO results	74		2.94 (0.44)		61		2.98 (0.43)		53			3.05 (0.49)		29			3.04 (0.55)		0.01 (0.08)			.88		–0.00 (0.08)			.97		0.00 (0.10)			.96	
	Return of PRO results + *Living with lymphoma*	76		2.97 (0.53)		64		2.95 (0.50)		58			2.98 (0.47)		35			2.94 (0.40)		-0.05 (0.07)			.53		–0.05 (0.08)			.50		–0.11 (0.09)			.22	
**HeiQ constructive attitudes and approaches (*P*=.36^b^)**																																		
	CAU	76		3.26 (0.52)		62		3.16 (0.51)		55			3.31 (0.50)		34			3.29 (0.47)		N/A			N/A		N/A			N/A		N/A			N/A	
	Return of PRO results	74		3.27 (0.51)		61		3.30 (0.49)		53			3.35 (0.44)		29			3.26 (0.60)		0.13 (0.07)			.06		–0.01 (0.07)			.93		0.03 (0.09)			.73	
	Return of PRO results + *Living with lymphoma*	76		3.33 (0.43)		64		3.30 (0.49)		58			3.31 (0.45)		35			3.34 (0.44)		0.07 (0.07)			.28		–0.06 (0.07)			.41		–0.06 (0.09)			.52	
**HeiQ self-monitoring and insight (*P*=.82^b^)**																																		
	CAU	77		3.08 (0.45)		62		3.17 (0.32)		55			3.15 (0.40)		34			3.04 (0.53)		N/A			N/A		N/A			N/A		N/A			N/A	
	Return of PRO results	74		3.05 (0.39)		62		3.11 (0.40)		53			3.13 (0.42)		29			3.14 (0.35)		0.06 (0.07)			.36		–0.02 (0.07)			.78		0.09 (0.09)			.31	
	Return of PRO results + *Living with lymphoma*	76		3.00 (0.42)		64		3.05 (0.38)		59			3.11 (0.39)		35			3.05 (0.40)		0.07 (0.07)			.30		0.02 (0.07)			.75		0.08 (0.08)			.33	
**HeiQ health services navigation (*P*=.26^b^)**																																		
	CAU	76		3.30 (0.45)		62		3.22 (0.44)		55			3.30 (0.47)		34			3.29 (0.42)		N/A			N/A		N/A			N/A		N/A			N/A	
	Return of PRO results	74		3.28 (0.39)		61		3.26 (0.40)		53			3.38 (0.43)		29			3.33 (0.44)		0.06 (0.06)			.35		0.07 (0.07)			.30		0.04 (0.08)			.61	
	Return of PRO results + *Living with lymphoma*	76		3.22 (0.49)		64		3.19 (0.50)		58			3.22 (0.41)		35			3.15 (0.36)		0.05 (0.06)			.40		0.02 (0.07)			.75		–0.13 (0.08)			.11	
**HeiQ social integration and support (*P*=.68^b^)**																																		
	CAU	76		3.15 (0.57)		62		3.07 (0.54)		55			3.12 (0.51)		34			3.13 (0.50)		N/A			N/A		N/A			N/A		N/A			N/A	
	Return of PRO results	74		3.12 (0.46)		61		3.13 (0.50)		53			3.15 (0.47)		29			3.16 (0.41)		0.06 (0.07)			.38		0.02 (0.07)			.82		0.04 (0.090)			.65	
	Return of PRO results + *Living with lymphoma*	76		3.22 (0.47)		64		3.20 (0.46)		58			3.16 (0.49)		35			3.19 (0.38)		0.06 (0.07)			.38		-0.02 (0.07)			.76		–0.08 (0.09)			.33	
**HeiQ emotional wellbeing (*P*=.90^b^)**																																		
	CAU	76		1.82 (0.62)		62		1.88 (0.65)		55			1.72 (0.55)		34			1.80 (0.50)		N/A			N/A		N/A			N/A		N/A			N/A	
	Return of PRO results	74		1.83 (0.52)		62		1.82 (0.53)		53			1.73 (0.53)		29			1.88 (0.68)		–0.06 (0.07)			.45		0.02 (0.08)			.76		–0.02 (0.10)			.81	
	Return of PRO results + *Living with lymphoma*	76		1.71 (0.48)		64		1.72 (0.51)		59			1.65 (0.49)		35			1.64 (0.53)		–0.05 (0.07)			.53		0.02 (0.08)			.82		–0.08 (0.09)			.36	
**Satisfaction with information provision (ISF^g^ total information provision; *P*=.66^b^)**																																		
	CAU	76		3.86 (0.78)		63		3.73 (0.79)		57			3.88 (0.76)		34			3.85 (0.82)		N/A			N/A		N/A			N/A		N/A			N/A	
	Return of PRO results	74		3.91 (0.72)		62		3.95 (0.71)		53			4.02 (0.57)		28			3.79 (0.79)		0.19 (0.13)			.14		0.10 (0.13)			.44		–0.01 (0.16)			.97	
	Return of PRO results + *Living with lymphoma*	76		3.86 (0.80)		65		3.68 (0.94)		59			3.80 (0.76)		35			3.80 (0.68)		–0.02 (0.12)			.88		–0.02 (0.13)			.85		0.02 (0.15)			.92	
**Health care use (contacts with general practitioner in past 12 months; *P*=.17^b^)**																																		
	CAU	74		4.70 (3.92)		63		3.16 (2.57)		57			3.63 (4.51)		34			3.38 (3.09)		N/A			N/A		N/A			N/A		N/A			N/A	
	Return of PRO results	74		4.78 (4.17)		62		4.40 (4.97)		53			3.68 (5.27)		29			4.90 (6.21)		1.04 (0.66)			.11		–0.15 (0.69)			.83		0.29 (0.85)			.73	
	Return of PRO results + *Living with lymphoma*	76		4.89 (4.32)		65		3.34 (3.25)		59			3.17 (3.24)		35			2.57 (3.00)		–0.16 (0.65)			.81		–0.97 (0.68)			.15		–1.45 (0.82)			.08	
**Contacts with medical specialist in past 12 months (*P*=.80^b^)**																																		
	CAU	76		10.09 (6.27)		63		7.00 (4.12)		56			6.66 (5.92)		34			5.44 (4.54)		N/A			N/A		N/A			N/A		N/A			N/A	
	Return of PRO results	74		10.42 (6.72)		61		6.70 (4.73)		53			5.70 (5.54)		29			5.21 (5.84)		–0.33 (1.10)			.77		–1.20 (1.15)			.30		–1.59 (1.41)			.26	
	Return of PRO results + *Living with lymphoma*	74		10.46 (7.18)		63		7.30 (5.43)		58			5.97 (3.79)		35			5.26 (6.34)		0.18 (1.10)			.87		–1.05 (1.14)			.35		–1.52 (1.36)			.26	

^a^HADS: Hospital Anxiety and Depression Scale (psychological distress subscale range, 0-42, with higher scores indicating more psychological distress).

^b^*P* value of the overall time-by-group interaction.

^c^CAU: care as usual (control group; reference category).

^d^N/A: not applicable.

^e^PRO: patient-reported outcome (T0, baseline assessment; T1, short-term follow-up assessment at 4 months postrandomization; T2, follow-up assessment at 12 months postrandomization; T3, follow-up assessment at 24 months postrandomization).

^f^HeiQ: Health Education Impact Questionnaire (self-management ability subscales range, 0-4, with higher scores indicating higher levels of self-management ability).

^g^ISF: Information Satisfaction Questionnaire (information satisfaction subscale range, 0-5, with higher scores indicating more satisfaction with perceived information).

Of the 150 patients who were randomized to arm 2 or 3 and had access to individual PRO results, 115 (76.7%) patients viewed their PRO results. The majority (79/115, 68.7%) viewed their PRO results more than once, and 16% (13/79) viewed it more than 5 times.

Patients with lymphoma who viewed their PRO results were more recently diagnosed (13.6 vs 15.0 months; *P*=.03), had a more conscientious personality (3.8 vs 3.6; *P*=.01), and had a less fatalistic coping style than those who did not (2.1 vs 2.2; *P*=.02; Table 3). In addition, patients who did view their PRO results more often wished to receive all available information about the disease compared with those who did not (68/115, 59.1% vs 10/35, 29%; *P*=.004).

Of those who viewed the PRO results, 91.3% (105/115) wished to compare their individual results to other patients with lymphoma and 80.8% (93/115) to a normative population without cancer. Only 7.8% (9/115) solely wanted to view their individual results.

**Table 3:** Baseline characteristics of patients who viewed their individual patient-reported outcome (PRO) results and those who did not.

				Those who viewed their PRO results (N=115)		Those who did not view their PRO results (N=35)		*P* value^a^	
**Sociodemographic characteristics**									
	Age at time of questionnaire (years), mean (SD)			60.1 (13.6)		61.4 (14.0)		.64	
	**Sex, n (%)**								.93
		Male	83 (72.2)		25 (71.4)				
		Female	32 (27.8)		10 (28.6)				
	**Educational level^b^, n (%)**								.16
		Low	4 (3.5)		1 (2.9)				
		Medium	51 (44.3)		22 (62.9)				
		High	60 (52.2)		12 (34.3)				
	Partner (yes), n (%)			14 (12.2)		8 (22.9)		.12	
**Clinical characteristics**									
	Months since diagnosis, mean (SD)			13.6 (3.3)		15.0 (3.3)		.03	
	**Cancer type, n (%)**								.21
		Hodgkin lymphoma	10 (8.7)		7 (20)				
		NHL-HG^c^	67 (58.3)		16 (45.7)				
		NHL-LG^d^	30 (26.1)		8 (22.9)				
		CLL^e^	8 (6.9)		4 (11.4)				
	**Treatment, n (%)**								.63
		Active treatment received	91 (79.1)		29 (82.9)				
		Active surveillance	24 (20.9)		6 (17.1)				
	Number of self-reported comorbidities, mean (SD)			1.1 (1.1)		1.1 (1.2)		.94	
**Psychological characteristics**									
	**Personality, mean (SD)**								
		Openness	3.5 (0.6)		3.4 (0.6)		.43		
		Conscientiousness	3.8 (0.4)		3.6 (0.5)		.01		
		Extraversion	3.6 (0.6)		3.5 (0.5)		.66		
		Agreeableness	3.8 (0.4)		3.7 (0.4)		.14		
		Neuroticism	2.4 (0.6)		2.4 (0.6)		.78		
	**Coping strategies, mean (SD)**								
		Fighting spirit	3.0 (0.4)		3.0 (0.4)		.47		
		Anxious preoccupation	2.3 (0.4)		2.2 (0.4)		.07		
		Helplessness or hopelessness	1.6 (0.4)		1.6 (0.5)		.87		
		Fatalism	2.1 (0.4)		2.2 (0.3)		.02		
		Avoidance	1.7 (0.7)		1.6 (0.6)		.47		
	Psychological distress (yes), n (%)			101 (87.8)		32 (91.4)		.56	
	Self-monitoring and insight, mean (SD)			3.1 (0.4)		2.9 (0.4)		.01	
	Satisfaction with information provision, mean (SD)			3.9 (0.8)		3.9 (0.7)		.76	

^a^*P* reports comparisons according to analysis of variance and chi-square tests.

^b^Educational levels were defined as follows: low=none or primary school; medium=lower general secondary education or vocational training; or high=preuniversity education or high-level vocational training or university.

^c^NHL-HG: high-grade non-Hodgkin lymphoma.

^d^NHL-LG: low-grade non-Hodgkin lymphoma.

^e^CLL: chronic lymphocytic leukemia.

## Discussion

### Principal Findings

The results of this RCT demonstrated that patients were neither in a better nor in a worse situation when their individual PRO results were disclosed, as no effects of return of PRO results were found on psychological distress, self-management skills, and satisfaction with information provision. In addition, patients who received their PRO results did not report more contact with their general practitioner or medical specialist compared with those receiving CAU.

Return of individual PRO research results seems to meet patients’ wishes, as the majority of those with access viewed their individual results, of whom two-thirds viewed it more than once. The possibility of comparing their scores with peers was most often chosen, indicating the importance of including normative data to place outcomes in perspective. Almost a quarter chose not to receive their results. Therefore, patients should have the choice as to whether they would like to receive their outcomes [14].

We observed little statistical differences in characteristics of patients who did view their PRO results and those who did not. Patients who viewed their PRO results more frequently wanted to receive all available information about the disease compared with those who did not view their results. This is in line with the literature that patients with a monitoring (information seeking) coping style tend to benefit from more provided information, whereas patients with a blunting (information avoiding) coping style benefit from less information [48,49].

There is increasing interest in integrating the collection of PROs in routine practice to enhance clinical care [50]. Weekly measurement of symptoms by patients during active treatment has proven to be effective, as 2 landmark studies on advanced solid tumors showed improvement in HRQoL and survival [51-53]. In our RCT, return of PRO results took place after treatment completion, including patients without advanced cancer stage, not specifically focusing on symptoms and was returned to patients only and not to their treating physician, which all may have contributed to differences in effects. With the continuing development of new and often expensive therapies for patients with cancer (eg, immunotherapy), further research and monitoring of the early onset and course of symptoms remains highly needed [54].

We could not compare our results to the evidence-based BREATH intervention for breast cancer [32,33], the original web-based self-management intervention from which Living with lymphoma intervention was derived, as uptake in our RCT was too low to study the effect. With respect to the design of the study, it was our explicit intention to examine the uptake and effects of a self-management intervention (without personal contact with a therapist) in a population-based setting (where all patients were asked, and no screening took place) to evaluate if this would be a possibility to provide low-intensity care to the continuously growing group of cancer survivors. This may have contributed to the much lower uptake compared with that of BREATH. Therapist guidance may improve patient engagement with a self-management intervention [55]. An important aspect of engagement is self-efficacy, which is a central element of several therapies, such as behavior change theory and planned behavior as a health access approach.

With respect to the *Living with lymphoma* intervention, we were not able to determine an effect as uptake was too low (3/76, 4%). Only 2 patients opened several components of the cognitive behavioral therapy parts, and various items from the library were viewed: *Reliable information*, *Fatigue*, *Emotional counselling*, and *Nutrition and cancer*, *Exercise*, *Physical counselling*, *Sexuality*, and *Reintegration*. As we observed that adherence was very low, we asked patients about reasons for nonadherence, and they indicated they felt well and still had regular appointments with their hematologist and therefore were not in need of an intervention. Furthermore, the majority of participants were men, and poor engagement in self-management was more common for men, as men may be more reluctant to seek help [56]. In addition, the timing of the intervention may not have been optimal, as participants were on average 14 months after diagnosis and patients appeared more receptive to interventions offered near diagnosis [57]. There is evidence that unguided self-management interventions could be more effective when targeted to those in the greatest need of an intervention, such as patients with low distress [32,58]. This might suggest that the need for intervention in our sample may be low and we may have not reached the right group, despite the invitation of a population-based sample and limited exclusion criteria.

This is the first RCT to study the effect of the return of individual PRO results to patients with lymphoma or to patients with cancer in general. The strengths of this study include the design and linkage of PROs with clinical data from the NCR. Owing to the design of our RCT, within a population-based observational cohort, we had access to sociodemographic information about nonparticipants. This provides a unique opportunity to make clear statements about the representativeness of the sample. Participants of the RCT were younger, more often male, and more often highly educated [59]. Ideally, the RCT sample will be representative of the entire target population so that generalizations about the population can be made. More research is needed to understand why underrepresented patients were not reached and how they could be reached in the future.

### Conclusions

In conclusion, the return of individual PRO results seems to meet patients’ wishes, even though it had no beneficial effects on patient outcomes; it did not have negative effects either. Therefore, we decided to include and implement the return of individual PRO results in the PROFILES registry. In addition, at this moment, the return of individual PRO results is extended to other cancer types, such as colorectal cancer, and is used in daily clinical practice. No conclusions could be drawn about the effectiveness of the self-management intervention because the uptake was too limited.

## Appendix

Multimedia Appendix 1 CONSORT-eHEALTH checklist (V 1.6.1).

## Abbreviations

BREATH: Breast Cancer eHealth
CAU: care as usual
CONSORT: Consolidated Standards of Reporting Trials
ES: effect size
HRQoL: health-related quality of life
LIVE: Lymphoma Intervention
NCR: Netherlands Cancer Registry
PRO: patient-reported outcome
PROFILES: Patient-Reported Outcomes Following Initial Treatment and Long-Term Evaluation of Survivorship
RCT: randomized controlled trial
